# Translation of Nutritional Genomics into Nutrition Practice: The Next Step

**DOI:** 10.3390/nu9040366

**Published:** 2017-04-06

**Authors:** Chiara Murgia, Melissa M. Adamski

**Affiliations:** Department of Nutrition, Dietetics and Food, Monash University, Notting Hill, VIC 3168, Australia; melissa.adamski@monash.edu

**Keywords:** nutrigenetics, nutritional genomics, dietetics, best-practice

## Abstract

Genetics is an important piece of every individual health puzzle. The completion of the Human Genome Project sequence has deeply changed the research of life sciences including nutrition. The analysis of the genome is already part of clinical care in oncology, pharmacology, infectious disease and, rare and undiagnosed diseases. The implications of genetic variations in shaping individual nutritional requirements have been recognised and conclusively proven, yet routine use of genetic information in nutrition and dietetics practice is still far from being implemented. This article sets out the path that needs to be taken to build a framework to translate gene–nutrient interaction studies into best-practice guidelines, providing tools that health professionals can use to understand whether genetic variation affects nutritional requirements in their daily clinical practice.

It is common for nutrition professionals to experience that the same dietary intervention and management strategy produce very different outcomes in different people. An overwhelming number of observations support the evidence that genetic background has a key role to play in individual response to diet and life-style, and in shaping individual nutritional requirements [1]. Proof of concept of these statements came from the well-described examples of inborn metabolic syndromes caused by single gene mutations affecting specific metabolic pathways. These syndromes are often successfully controlled with targeted dietary management which is able to prevent serious health consequences [2].

Phenylketonuria (PKU) is a rare inborn syndrome caused by a mutation in a single gene that encodes for the enzyme phenylalanine hydroxylase. The liver of PKU mutation carriers in homozygosis are unable to break down phenylalanine and are consequently unable to metabolise food that contains this amino acid. PKU was one of the first genetic conditions to be identified, and it is the result of a mutation in gene coding for an enzyme involved in a key step of a metabolic pathway; to date, the only effective management for PKU patients is a carefully tailored low protein diet [3]. Common polymorphisms (frequency > 1%) can also determine dietary requirements, for example, lactose intolerance is caused by the progressive reduction of the expression of the gene coding for the enzyme lactase due to a variant in the regulatory region of the gene. Carriers of these high frequency variants develop adverse symptoms if they consume milk or other lactose-rich dairy products [4]. These very well characterised examples show how the idea of changing, even dramatically, the diet of individuals carrying specific genetic variants is a common dietetic practice; so is the knowledge that foods, such as milk, that are highly nutritious for some, need to be consumed with care by others. The examples reported are relatively simple: a specific nutritional requirement is the consequence of a mutation or variation in a single gene. However, most of the time, the reality of nutrition and dietetic practice is more complicated. The metabolism of each nutrient involves the activity of several enzymes, each one encoded by a gene that is present in the population with multiple allelic variants; each of them potentially contributing to the absorption and utilisation of nutrients, ultimately affecting their requirements [5,6]. More than one genetic polymorphism is generally responsible for affecting the requirements of a nutrient or the predisposition to a chronic pathology. Indeed, it is now evident that even in traditionally classified monogenic conditions such as PKU, the penetrance and severity of the symptoms are determined by other gene variants, each one contributing with a specific effect size; in real life, every phenotype is produced by a combination of gene variants [3].

The completion of the Human Genome Project (HGP) [7] held the promise of resolving the complexity of individual responses to diet and the sometimes ineffectiveness of “one size fits all” public health guidelines for individuals, revealing the role of genetics in shaping nutritional requirements. The potential applications to nutrition of this invaluable tool were apparent since the genome was mapped. The first articles discussing nutrigenomics and nutrigenetics were published less than a year after the first draft and an initial analysis of the human DNA sequence was made available. Several papers discussed the potential impact of this new information on nutrition practice [7,8,9]. Since then, many authors have outlined how this new area of science may impact on nutrition practice. They have discussed what would be needed in terms of training and required knowledge for nutrition professionals, potential ethical, legal and social impacts on practice and other impacts on nutrition [10,11,12]. However, fifteen years and hundreds of publications later, the gap between the experimental and epidemiologic evidence and health practice is not yet closed [13]. The information provided by the HGP and the resources developed since its publication were applied in hundreds of studies; a PubMed search performed on 16 March 2017, using the search term “nutrigenomics OR nutrigenetics OR nutritional genomics” resulted in 2888 hits. While these papers/research are essential for understanding the impact of this new information on nutrition science and identifying what is needed to successfully put it into practice, progress in actually interpreting/translating the current evidence into useful information for healthcare professionals has yet to be seen. The importance of the genotype information is not the only factor that complicates this translation into practice; the discovery of other levels of control to dietary phenotyping, including environment-modulated epigenome and the intestinal microbiome are other complicating factors [14,15].

To translate genetic information into evidence-based nutritional recommendations, clear summaries and critical analysis of the current evidence base of identified polymorphisms and their interaction with nutrition and health need to be provided, including their effect size. This will allow healthcare professionals to more easily understand the relationship with nutrient metabolism, and whether there is any robust evidence for an intervention. First, the results in systematic reviews and meta-analysis need to be collated and second, user-friendly computational tools that can integrate the complex genetic and epigenetics information need to be built, integrating it with clinical and biochemical biomarkers. This approach has achieved a promising step toward with Eran Segal’s group at Weizemann Institute. This important study developed an algorithm that integrates blood parameters, dietary habits, anthropometrics, physical activity, and gut microbiota to predict blood glucose response to meals with different contents of carbohydrates [16]. However, this complex tool does not take genetics or epigenetics into consideration. 

A limited number of papers have reviewed the evidence in regards to specific areas of nutrigenetics in an attempt to collate and build the literature base [6,17,18,19]. This is one step closer to the inclusion of genetic variability as an important component and the translation of evidence into practice—but it is only the beginning.

Health professionals require clear best-practice guidelines to be able to translate the evidence into nutrition advice. In general, to guide nutrition and dietetic practice, nutrition research is regularly reviewed and analysed by experts to provide summaries of the latest evidence to guide practice. For example, the National Health and Medical Research Council in Australia provides guidance for the recommended nutrient intake along with summaries of the latest available evidence [20]. These are updated by expert working groups at regular periods to ensure up-to-dateness. Other countries also conduct similar research and translation [21,22]. These guidelines assist nutrition professionals in providing current, evidence-based recommendations. Similar processes occur in other health disciplines, where evidenced-based guidelines are developed, and regularly reviewed and updated to guide healthcare and medical professionals’ practice. 

Nutritionists and dietitians were identified as prime candidates to provide advice on nutrition and genetics, but the development of guidelines and evidence summaries for these professionals has been scarce [23]. Currently, nutrition professionals are expected to search, critically analyse and translate the findings into practice; for many, this may provide a challenge, especially since extensive genetic and genomic training is not a traditional area of training for nutrition professionals [24,25]. Assessments of the literature in regards to gene–nutrient interactions, similar to the Dietary Reference Intakes (DRIs) (USA) and Nutrient Reference Values (NRVs) (Australia), must be performed in order to translate nutritional genomics into clinical practice. We need to use the experience to build a framework to translate gene–nutrient interaction studies into usable guidelines and provide answers to nutrition professionals looking to understand whether genetic variation affects nutritional requirements. These assessments of the literature should be carried out by expert working groups comprising of geneticists, bioinformaticians, nutritionists, dietitians and clinical geneticists.

While the science of nutritional genomics continues to demonstrate potential individual responses to nutrition, the complex nature of gene, nutrition and health interactions continues to provide a challenge for healthcare professionals to analyse, interpret and apply to patient recommendations. Evidenced-based summaries and interpretations of the current literature base urgently need to be developed by expert working teams of both genetic experts and nutrition experts. These summaries can then provide the basis for the development of best-practice guidelines for nutrition professionals for use in clinical practice.

## References

[1] Fenech M., El-Sohemy A., Cahill L., Ferguson L.R., French T.A., Tai E.S., Milner J., Koh W.P., Xie L., Zucker M. (2011). Nutrigenetics and nutrigenomics: Viewpoints on the current status and applications in nutrition research and practice. J. Nutrigenet. Nutr..

[2] Boyer S.W., Barclay L.J., Burrage L.C. (2015). Inherited metabolic disorders: Aspects of chronic nutrition management. Nutr. Clin. Pract..

[3] Scriver C.R. (2007). The pah gene, phenylketonuria, and a paradigm shift. Hum. Mutat..

[4] Deng Y., Misselwitz B., Dai N., Fox M. (2015). Lactose intolerance in adults: Biological mechanism and dietary management. Nutrients.

[5] Frazier-Wood A.C. (2015). Dietary patterns, genes, and health: Challenges and obstacles to be overcome. Curr. Nutr. Rep..

[6] Day K.J., Adamski M.M., Dordevic A.L., Murgia C. (2017). Genetic variations as modifying factors to dietary zinc requirements—A systematic review. Nutrients.

[7] Lander E.S., Linton L.M., Birren B., Nusbaum C., Zody M.C., Baldwin J., Devon K., Dewar K., Doyle M., FitzHugh W. (2001). Initial sequencing and analysis of the human genome. Nature.

[8] Elliott R., Ong T.J. (2002). Nutritional genomics. BMJ-Brit. Med. J..

[9] Van Ommen B., Stierum R. (2002). Nutrigenomics: Exploiting systems biology in the nutrition and health arena. Curr. Opin. Biotech..

[10] DeBusk R. (2009). Diet-related disease, nutritional genomics, and food and nutrition professionals. J. Am. Diet. Assoc..

[11] DeBusk R.M., Fogarty C.P., Ordovas J.M., Kornman K.S. (2005). Nutritional genomics in practice: Where do we begin?. J. Am. Diet. Assoc..

[12] Rosen R., Earthman C., Marquart L., Reicks M. (2006). Continuing education needs of registered dietitians regarding nutrigenomics. J. Am. Diet. Assoc..

[13] Ferguson J.F., Allayee H., Gerszten R.E., Ideraabdullah F., Kris-Etherton P.M., Ordovas J.M., Rimm E.B., Wang T.J., Bennett B.J. (2016). Nutrigenomics, the Microbiome, and Gene-Environment Interactions: New Directions in Cardiovascular Disease Research, Prevention, and Treatment. Circ. Cardiovasc. Genet..

[14] Dey M. (2017). Toward a personalized approach in prebiotics research. Nutrients.

[15] Sharma U., Rando O.J. (2017). Metabolic inputs into the epigenome. Cell Metab..

[16] Zeevi D., Korem T., Zmora N., Israeli D., Rothschild D., Weinberger A., Ben-Yacov O., Lador D., Avnit-Sagi T., Lotan-Pompan M. (2015). Personalized nutrition by prediction of glycemic responses. Cell.

[17] Kanoni S., Nettleton J.A., Hivert M.F., Ye Z., van Rooij F.J., Shungin D., Sonestedt E., Ngwa J.S., Wojczynski M.K., Lemaitre R.N. (2011). Total zinc intake may modify the glucose-raising effect of a zinc transporter (SLC30A8) variant: A 14-cohort meta-analysis. Diabetes.

[18] Shaghaghi M.A., Kloss O., Eck P. (2016). Genetic variation in human vitamin c transporter genes in common complex diseases. Adv. Nutr..

[19] Colson N.J., Naug H.L., Nikbakht E., Zhang P., McCormack J. (2017). The impact of mthfr 677 C/Tgenotypes on folate status markers: A meta-analysis of folic acid intervention studies. Eur. J. Nutr..

[20] Nutrient Reference Values. https://www.nrv.gov.au/.

[21] Food and Nutrition Board, Institute of Medicine (I.o.M). http://nationalacademies.org/hmd/about-hmd/leadership-staff/hmd-staff-leadership-boards/food-and-nutrition-board.aspx.

[22] Scientific Advisory Committee on Nutrition (SACN), U.K.G.. https://www.gov.uk/government/groups/scientific-advisory-committee-on-nutrition.

[23] Kicklighter J.R., Dorner B., Hunter A.M., Kyle M., Pflugh Prescott M., Roberts S., Spear B., Hand R.K., Byrne C. (2017). Visioning report 2017: A preferred path forward for the nutrition and dietetics profession. J. Acad. Nutr. Diet..

[24] Collins J., Bertrand B., Hayes V., Li S.X., Thomas J., Truby H., Whelan K. (2013). The application of genetics and nutritional genomics in practice: An international survey of knowledge, involvement and confidence among dietitians in the US, Australia and the UK. Genes Nutr..

[25] Li S.X., Collins J., Lawson S., Thomas J., Truby H., Whelan K., Palermo C. (2014). A preliminary qualitative exploration of dietitians’ engagement with genetics and nutritional genomics: Perspectives from international leaders. J. Allied Health.

